# The Open Anatomy Browser: A Collaborative Web-Based Viewer for Interoperable Anatomy Atlases

**DOI:** 10.3389/fninf.2017.00022

**Published:** 2017-03-27

**Authors:** Michael Halle, Valentin Demeusy, Ron Kikinis

**Affiliations:** ^1^Surgical Planning Laboratory, Department of Radiology, Brigham and Women’s Hospital, Harvard Medical SchoolBoston, MA, USA; ^2^Mathematics - Computing and Information Technology, Ecole Centrale LyonLyon, France; ^3^Fraunhofer MEVISBremen, Germany

**Keywords:** anatomy atlases, visualization, WebGL, open-source software, open data, format standardization, collaborative software

## Abstract

The Open Anatomy Browser (OABrowser) is an open source, web-based, zero-installation anatomy atlas viewer based on current web browser technologies and evolving anatomy atlas interoperability standards. OABrowser displays three-dimensional anatomical models, image cross-sections of labeled structures and source radiological imaging, and a text-based hierarchy of structures. The viewer includes novel collaborative tools: users can save bookmarks of atlas views for later access and exchange those bookmarks with other users, and dynamic shared views allow groups of users can participate in a collaborative interactive atlas viewing session. We have published several anatomy atlases (an MRI-derived brain atlas and atlases of other parts of the anatomy) to demonstrate OABrowser’s functionality. The atlas source data, processing tools, and the source for OABrowser are freely available through GitHub and are distributed under a liberal open source license.

## Introduction

Anatomy atlases are a visual representations of medical knowledge, and as such are useful to a wide range of users, including physicians, medical students, researchers and the general public. Many different anatomy atlases exist, both in print and digital forms. Each has their strengths, driven by their underlying medical expertise, the type and quality of presentation they offer (e.g., two-dimensional drawings, three-dimensional renditions, or detailed anatomical data), and their target audience. The neuroscience research community relies heavily on atlas data as a reference for ongoing research studies. The community has implemented organizational tools such as the Neuroscience Information Framework (NIF) and the Neuroimaging Informatics Tools and Resources Clearinghouse (NITRC) to provide catalogs of atlases, atlas tools and ontologies for organizing and processing neuroanatomical information.

Our focus is on improving the dissemination, presentation and collaborative potential of the visual information contained in anatomy atlases for practical use by a wide audience. There are dozens of examples of existing visual anatomy browsers. Many of these viewing tools are closely coupled to a particular atlas data set or research effort (for example, the Allen Brain Map and BrainExplorer; Sunkin et al., 2013). Close coupling assures that viewing tools work well with a particular atlas. However, it also limits the resources available to, say, create a new viewer designed to address the needs of a different audience. Ideally, atlases and atlas viewers (and other atlas processing tools) could be decoupled from each other so that high quality tools could be used interoperably with different atlases. This goal of interoperability has been expressed clearly in the work of the International Neuroinformatics Coordinating Facility’s (INCF) Digital Atlasing effort (Hawrylycz et al., 2011).

The Scalable Brain Atlas (Bakker et al., 2015), a software platform developed as part of the INCF, attempts to address this atlas interoperability problem by providing a set of standardized data and tools for “all publicly available brain atlases that are of sufficient interest to the (neuroscience) community”. The Scalable Brain Atlas consists of processed versions of approximately 20 human and animal brain atlases, web services that allow uniform access to atlas information, and a web-based viewer for displaying two-dimensional cross-sections and three-dimensional rendered views of the atlases. The Scalable Brain Atlas source code is open source distributed under the GNU Public License (GPL)^1^.

The Scalable Brain Atlas is organized as a centralized model: atlases are imported into the framework, converted into a common form, and made available collectively through a monolithic web portal, including 2D and 3D atlas viewing tools. While a centralized development model can produce consistent, well-organized results, it places the burden of conversion, distribution and display on a single entity.

Recently, members of research teams from several anatomy atlas development groups comprising the Human Atlas Working Group (HAWG) met to develop an interoperability and exchange data format for anatomy atlases under the umbrella of INCF (HAWG, Human Atlasing Working Group, 2016)^2^. Such a format would allow atlases to share viewing tools, data editors and other atlas creation software, reducing duplication of effort. A common file data format encourages a decentralized community of atlas development, in contrast to the Scalable Brain Atlas’s centralized model. This decentralized development model would also allow “best of breed” or specialized tools to become widely used across many atlas projects, building community and simplifying user training. As members of HAWG, we have contributed our experience with internal data formats in 3D Slicer (Pieper et al., 2004), our open source medical image informatics and visualization platform, towards the standard development process.

While the HAWG standardization efforts have their genesis in neuroscience, a flexible atlas interchange format has clear utility for the broader atlas community as well. Many other medical specialties have ongoing atlas efforts throughout the human anatomy as well as in a variety of other organisms. In addition, commercial entities and some research groups offer more general “whole body” atlases that target broad audiences including medical education and public outreach. An example of open and less specialized anatomy atlas development is BodyParts3D (Mitsuhashi et al., 2009). BodyParts3D is an ongoing open-source and open-data project to build 3D structural segments of a whole-body human male based on a 2 mm MRI scan of a volunteer. Medical concepts from the Foundational Model of Anatomy (FMA; Rosse and Mejino, 2003) ontology were used to define anatomical segments, which were then modeled by illustrators. The resulting models and systems are available to view online and for download for alternative purposes such as 3D printing.

Our work with digital interactive anatomy atlases dates more than 20 years (Shenton et al., 1995). We have produced several anatomy atlases consisting of source radiology images, segmented voxel maps labeling different anatomical structures, polygonal models of those structures, all arranged in a hierarchical organization. Examples of these atlases include an MRI-derived neuroanatomic atlas^3^ as well as knee^4^, abdominal^5^ and head and neck^6^ atlases based on CT imaging. Until recently, however, these atlases have been developed in a closed process by a small number of people, with only the resulting data made available.

Separate from the atlases themselves, we have also developed atlas viewing tools. Because our atlases are based on tomographic data, the viewers have the ability to display image slices and three-dimensional views of anatomic structures. These tools include one of the earliest web-based visual anatomy applications using Java (Golland et al., 1998) In recent years, we have primarily used 3D Slicer to author and view atlases, which prevents us from reaching all but the most dedicated members of the public.

The Open Anatomy Browser (OABrowser for short) described in this article is a web-browser based anatomy atlas viewer originally designed as a test bed for evaluating ongoing draft versions of the HAWG data format, and as a vehicle for exploring interoperability between atlases. We have adopted and extended the work of HAWG (specifically, the discussions and drafts from the January 2016 HAWG meeting in Boston) to produce a practical anatomy viewer application. Our technology approach is similar to BrainBrowser (Sherif et al., 2015), but with HAWG’s data format as a foundation for interoperability. Compared to BrainBrowser, we have chosen to tailor OABrowser more as a standalone application suitable for use by a wide audience. OABrowser also includes novel collaborative features such as globally-shared bookmarks and dynamic shared views.

The OABrowser is part of our effort to open our atlas development pipeline and encourage community participation and code reuse. OABrowser and our larger atlas efforts are funded through the Neuroimage Analysis Center (NAC), an NIH-sponsored biomedical resource center. Our anatomy atlases are used by various NAC internal research efforts and also serve as a way to disseminate the results of the Center’s biomedical research. While NAC is focused specifically on neuroimaging, our broader biomedical research has produced atlases of the head and neck, abdomen and knee.

With NAC’s charter of dissemination, and based on our success in developing free and open source medical software (Pieper et al., 2006; Kapur et al., 2012), we have chosen a development philosophy emphasizing freely available tools and an open development process for creating shared anatomy atlases and atlas software. Our code, data and tools are licensed under the 3D Slicer open source license^7^, which allows for liberal use, reuse, and redistribution for any purpose, including commercial applications. Furthermore, OABrowser is designed to be as accessible as possible to a broad community, including the general public.

In order to test OABrowser, we have converted several of our imaging-derived anatomy atlases to draft HAWG data format and made the resulting atlases available for viewing. We have also done initial work to convert third party atlases for viewing using the atlas browser.

## Methods

**Figure 1:**
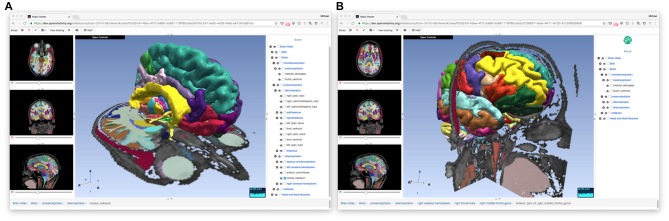
**Open Anatomy Browser (OABrower) showing different structures of the SPL brain atlas **(A,B)**.** The interface consists of a cross-sectional display panel on the left, a 3D structure view in the middle, and a text-based hierarchical structure view on the right. An information bar on the bottom displays the currently selected structure and its ancestors.

The OABrowser application is implemented as a single-page web application using current web standards. It is designed to work with three-dimensional anatomy atlases with volumetric imaging, geometric models and text metadata. The user interface consists of a cross-sectional slice panel on the left, a three-dimensional structure panel in the middle, and a text-based structure hierarchy on the right. The 3D display window shows geometric models of anatomic structures along with textured cross-sections of volumetric data, which are also displayed in the slice panel. An information bar lying along the bottom of the interface is used to display information about selected structures. Screenshots of OABrowser viewing the SPL/NAC brain atlas is shown in Figure 1.

The rest of this section describes the underlying software architecture of OABrowser.

### Software Technology Stack

OABrowser is implemented in the ES2015 dialect of JavaScript. It uses the Google-created Angular web framework^8^ and the AngularUI^9^ interface toolkit to manage the sub-windows, menus and application state. WebGL is used to display three-dimensional models of anatomy. WebGL is implemented on all major desktop and mobile platforms and web browsers, providing accelerated three-dimensional graphics and imaging capabilities to a large audience. HTML canvases are used for image display in the cross-sectional slice panel.

The higher-level JavaScript graphics library THREE.js^10^ abstracts low-level WebGL graphics calls. THREE.js includes loaders for common image and geometry file formats. We have extended THREE.js’s existing loaders to include better support for VTK files^11^ and the NRRD image file format^12^. These changes have been contributed back to THREE.js for that library’s community to use. THREE.js also provides mechanisms for selecting the three-dimensional structures under the user’s pointer, which OABrowser uses to select and provide information about anatomical structures.

### Atlas and Application State

The OABrowser architecture makes a clear distinction between atlas data and application state. Atlas data consists of image and geometry data that describes anatomical structures, annotation information that labels anatomical parts in a human readable way, and styles that depict how the structures should be displayed. Atlas data is stored in a standardized form to be described later in this article.

Application state describes which structures are selected, which slices are being displayed, the viewer’s point of view looking at the atlas, and any local modifications to appearance made by the user. Application state also includes a link to the atlas being viewed.

Application state is the core, centralized data structure in OABrowser. Application state can be stored and restored (for undo and redo), saved for later viewing (bookmarks), sent to another person or group (bookmark sharing), or shared dynamically (dynamic shared views). The application state data structure is indexed by unique identifier (known as a UUID^13^) and saved either in application memory or a persistent database. Given a UUID, OABrowser can look up its state information and restore itself to the given configuration.

For undo and redo, OABrowser using an in-memory internal stack to store application state. Every user interaction with the browser pushes a state object onto the stack. When the user clicks the browser’s back and forward buttons, for example, OABrowser restores the respective state object and updates the user interface accordingly.

OABrowser can also save application state to a centralized, world-readable networked database with a UUID key. Whenever the state of the user interface changes, the application state data object is saved away and a new one is created with a new UUID. This UUID is included in the browser’s URL string, which is updated as the user manipulates the atlas. At any one moment, then, the OABrowser URL contains enough information to load the application and recover sufficient application state to restore a viewing session. This URL is effectively a bookmark that allows the entire browser’s state to be restored.

OABrowser uses Firebase, a Google database and application framework, to implement shared application state. Firebase provides scalable real-time database services to web-browser based applications. It implements social logins from service providers such as Facebook, Google, GitHub and Twitter to allow users to write and edit their own bookmarks while allowing anyone to read them. While Firebase is a closed-source, commercial product, we are not fully dependent on it. Open source alternatives such as RethinkDB/Horizon^14^ or deepstream.io^15^ could be adapted to provide the same type of services as Firebase if needed.

In addition to traditional database services, Firebase broadcasts changes to entries in the database to interested web clients as they occur. Clients can use this information to update their internal application state. This feature allows multiple OABrowsers running across the network to synchronize, mirroring the appearance of the user interface for all users and allowing collaborative viewing and interaction. Firebase is part of the Google Cloud infrastructure, which assures sufficient capacity to support dynamic shared views between individuals, small teams, or in teacher-classroom settings.

### Data Format

**Figure 2:**
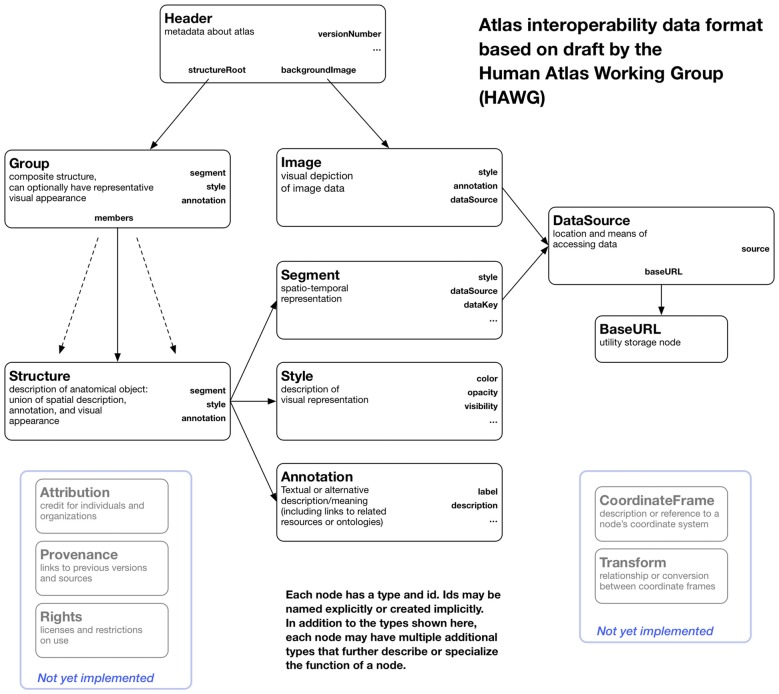
**A block diagram of the node structure of the data format used by OABrowser.** This format is based on early draft standards created by the Human Atlas Working Group (HAWG) to promote atlas interoperability and exchange. We have changed and extended the HAWG proposals as needed to create a working viewer application.

The atlas data itself is stored as files and web resources that can be distributed from any web server. The data consists of relatively large image and geometry data, more compact label map data, and lightweight metadata stored in a file format based on HAWG working drafts and extensions. We have added features to the HAWG format to permit multiple representations of atlas structures to be described (e.g., label maps and three-dimensional geometry), support for different file image and geometry file formats, and other pragmatic changes to build a working atlas tool. We have also changed some terminology for clarity, all while retaining basic concepts of the data format. Thus, our description of the format should be viewed as a proposal for a future draft release based on practical deployment experience. A block diagram of our variant of the HAWG format and our extensions are shown in Figure 2.

#### JSON and JSON-LD

HAWG files are written in a dialect of JSON (JSON, 2016) called JASON-LD (JSON-LD Specifications, 2016), which standardizes metadata and linked data conventions. Information about elements of the atlas is written as JSON-LD nodes, which are represented as JSON arrays. Attributes of nodes begin with a lower case letter by convention. Nodes in the HAWG data format have IDs that are unique within the file. Nodes can refer to other nodes using this ID. By convention, we have chosen to use IDs of the form “#idname” to mirror the common fragment identifier syntax of URIs. Indeed, JSON-LD encourages the use of URIs to identify resources, and this naming scheme allows us to uniquely reference nodes in external HAWG files. Nodes also have a type, capitalized by convention, that corresponds to a unique expanded URI-based type name in order to avoid name clashes and in the future allow access to nodes defined in multiple files.

The canonical form of a HAWG file is a JSON-LD compliant file where nodes are arrays in a JSON list, with references to node IDs connecting them together as needed. This canonical form is simple to parse, but it may not be simple to author. JSON-LD provides a variety of other forms, such as a nested hierarchy of nodes, and a well-defined mechanism to convert these forms into the canonical form. Furthermore, it is not strictly necessary to use a JSON-LD parser to parse the file format. A simple JSON parser with *ad hoc* rules for interpreting the data can also be used. At this time, OABrowser uses just such an *ad hoc* parser.

#### Structures and Groups

Anatomic parts are represented using Structures, with compound structures being represented as Groups. Structures point to other nodes that describe their spatial representation, their on-screen appearance, and their ontological label or other annotation. These elements are external to the structure itself to allow reuse of different parts of the atlas and to implement a separation of concerns for atlas authoring. For instance, new annotations can be added to the atlas or new colors or rendering styles can be assigned separately from the designation of segment data or geometry.

#### Header

Every atlas has a header (type Header) that provides information about the atlas itself and contains pointers to other atlas nodes. A Header node includes a “structureRoot” attribute that is a Group representing the root of the atlas hierarchy. Alternatively, the “structureRoot” attribute can have a JSON list value that includes more than one Group, in which case each Group in the list is considered to be a independent root. This feature allows multiple hierarchies of structures, possibly overlapping, to be described in a single file.

#### Segments

The spatial or spatio-temporal representation of a structure is represented as a Segment. Segments currently have two main subtypes: LabelMapSegment and GeometrySegment. A LabelMapSegment contains a volumetric description of the segment, while a GeometrySegment uses a polygonal model. A single Structure can have more than one Segment type, with each Segment node representing the Structure in a different way (say, both as a label map and as a polygonal model). Segments can be marked with a boolean attribute “authoritative” as true if it should be considered an authoritative spatial representation, while non-authoritative segments can be included for visual display where exact spatial boundaries might not be necessary.

In our atlases, the LabelMapSegment is authoritative to allow for slice overlays and interactive picking on slices, while non-authoritative GeometrySegments are used for polygonal models of anatomy and structure picking in 3D. This mechanism is an example of the kind of practical development that emerges only through actual application prototyping.

#### DataSource

The HAWG data format abstracts out the details of data access from the nodes that use the data. Data access is isolated in the DataSource node, which holds primary responsibility for describing data access through file names, URLs, or database queries. DataSource nodes, like all HAWG nodes, can be specialized by listing multiple types (For instance, a DataSource node that knows about remote files could be of type (“DataSource” “HTTPDataSource”). The use of types consisting of JSON lists is part of the JSON-LD standard and allows readers to function without implicit type information stored in a type hierarchy. Even naïve readers can distinguish DataSources from other node types, and more sophisticated ones can act differently on nodes that have more specialized subtypes.

It is anticipated that one DataSource might provide data to multiple Segments (if, say, a single file contains multiple image segments, in which case the key for selecting the correct segment is stored in the Segment itself and passed on to the DataSource).

#### Image

An Image node is similar in function to a Segment node but represents image data such as source CT or MRI scans. An Image node can be subtyped based on particular Image types, for instance (“Image” “NRRDImage”). Segment and Image nodes contain a reference to a DataSource node that they use to actually retrieve the data. Segment and Image nodes have provide enough parameter information to the DataSource to, for example, identify subsets or sub-dimensions of data.

A key property of the HAWG data format is its emphasis on representing metadata and incorporating external data such as images and geometry through reference. HAWG does not reinvent representations of concepts as coordinate systems or data representations, but instead references external files and data sources in already-established formats. This characteristic allows a tool such as OABrowser to be developed with a core set of supported data formats, with others added on as needed without any change in the underlying HAWG atlas description. OABrowser currently supports image and segmentation volumes in the NRRD file format and geometry descriptions stored in VTK, STL and Wavefront OBJ/MTL formats. We have chosen these formats because of their simplicity, their compatibility with our existing tools, and the availability of converters from other formats to them. We plan to support NIFTI and DICOM formats natively for images and segmentation objects in the future through a plugin architecture similar to BrainBrowser.

#### Annotation

Annotation nodes encapsulate human- or machine-readable annotation data describing structures. The simplest Annotation node, and the one currently implemented, includes a title and text description of a structure. Future Annotation subtypes may include references to controlled naming standards or links to ontologies. For example, structural information from the FMA ontology could be included in a (“Annotation” “FMAAnnotation”) type node. Multiple annotations can be applied to a structure, in which all annotations are considered valid alternative descriptions.

#### Style

Style nodes (formerly RenderOptions nodes in HAWG drafts) describe how a structure should be rendered visually. For example, vascular structures might be rendered red or blue, or various parts of the brain might be rendered in arbitrary but consistent colors. Multiple structures might share a single color if, say, the atlas renders by tissue or cell type. Style nodes provide the mechanism to perform these tasks by separating structure appearance from the structure itself, much in the same way cascading style sheets (CSS) separate style of web page elements from HTML document description. The content of Style nodes is currently very basic, including only color, transparency and visibility. We anticipate future, more complex subtypes of styles to take advantage of graphics features such as GPU-based shaders. We can also see a role for styles to describe the visual appearance of screen- or print-rendered annotation.

#### Attribution, Provenance and Rights

We also propose, but do not implement, several additional node types. The first set of nodes provide a mechanism to credit the authors and processes that have contributed to the creation of a node as well the conditions by which that node may be used. Attribution nodes associate human creators with nodes or groups of nodes. Provenance nodes identify the sources of information from which a node was derived. And the Rights node provides information on usage or copyright information of nodes in an atlas. These node types provide fine-grained attribution and prescription of atlas content in a way that provides both credit and accountability for individual elements of much scientific and creative works.

#### CoordinateFrame and Transform

Finally, we have proposed, but have not implemented, a simple mechanism to deal with simultaneous display of multiple atlases in different coordinate frames. During the HAWG discussions, an explicit decision was made to first address the description of a single atlas in a single coordinate frame to side step the issue of coordinate systems and handling. Coordinate systems are often specialized, domain-specific, or complicated. Duplicating existing coordinate frame description mechanisms or prematurely dictating one description scheme over another risks complexity and loss of generality.

Our mechanism is based on the observation that geometry and image file formats already have coordinate system descriptions that any DataSource, Segment, or Image designed for them already understands. We propose a CoordinateFrame node that merely points to a DataSource and provides enough information to specify a coordinate system described in that DataSource. Every node in the same coordinate system can point to a single CoordinateFrame. Another node type, a Transform node, as yet unspecified, describes the relationship between different CoordinateFrames and allows transformation between them. The (potentially composite) transformation between different CoordinateFrames can be discovered by inspecting the references between transforms and frames.

We look forward to further community discussion about the details of these proposed nodes.

### Revision Control and Data Store

While not strictly part of the OABrowser application, the workflow we use for managing software development, creating atlases and distributing atlas data is an important element that underlies the AOBrower user experience. In our current atlas development workflow, we use the git distributed revision control system^16^ and the GitHub collaborative code and data repository^17^ for both collaborative editing and distribution. The atlas viewer software is stored in a public GitHub repository. Master copies of the data for all of our atlases also reside on GitHub, where they are accessible to all. Through GitHub, users and other developers can offer modifications and file bug reports that can be used to improve OABrowser and our atlases.

For testing and modification, a developer can check out the latest “master” version (or any previous revision) of OABrowser or an atlas into a local repository on his or her computer. The atlas browser can view local data during development. Once changes and edits have been made, the developer commits in the local version to the master atlas repository. Clones of the GitHub atlas repositories can be copied to any standard web server as needed. We are actively testing deployment release versions of the atlas using distributed content networks such as Google Cloud infrastructure.

We currently use 3D Slicer as the primary authoring tool for our atlases. A python-based converter script extracts relevant atlas data and visual styles from Slicer’s internal file format MRML and writes it out in the HAWG-derived format. The script is stored as part of the atlas viewer’s source repository. We also have converter scripts to create HAWG files from simple TSV (tab-separated value) text tables.

### Licensing

The OABrowser software and sample atlases are licensed under the 3D Slicer License, which is based on the popular two-clause BSD license and includes some additional terms related to appropriate use in medical contexts. In particular, the Slicer License does not place any requirements on users or developers to release the source code of incorporating applications, in contrast to other licenses such as the GPL. We have found these licensing terms simplify collaboration with and dissemination to a wide range of academic and corporate institutions.

## Results

OABrowser’s original purpose was to validate the HAWG atlas data format and make our existing anatomy atlases available to the public through an ordinary web browser. We have been able to accomplish these goals and further gain new experience by implementing collaborative features made possible through the use of contemporary web technologies. We explain our results by first explaining the basic features of OABrowser, followed by our experience with HAWG-based atlas creation, and concluding with a look at performance and current deployment issues.

### Features

Inside OABrowser, anatomical structures can be inspected as 2D cross sections (in the slice panel or in the 3D view), as 3D models, or as entries in the structural hierarchy view. When a structure is selected, its name is displayed in the bottom status window, along with a “breadcrumb” list of parent and ancestor structures all the way up to the root of the atlas. Each ancestor name is a hyperlink that selects the respective containing structure.

A user can manipulate the slices and 3D views in OABrowser by panning, zooming and selecting structures. Structures appear in similar colors in the 3D view and in color overlays in the slices. Individual structures and trees of substructures can be turned on and off. Optional crosshairs indicate the location of cross-sectional slices in each view. The window and level of the volumetric data can be changed to improve image contrast. Ranges of voxel values can be rendered transparent to improve model visibility.

#### Bookmarks

**Figure 3:**
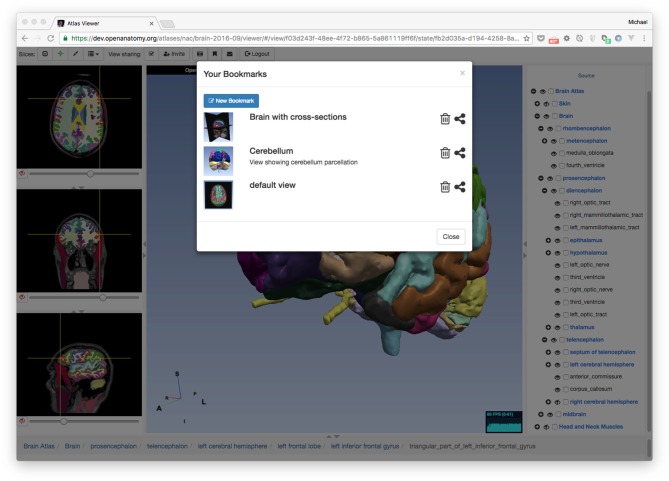
**Bookmarks.** OABrowser’s bookmark panel shows saved scene bookmarks. Bookmarks can be shared with other users. A user’s bookmarks may refer to any atlas. If the bookmarked atlas is not currently being viewed, OABrowser will offer to load it.

Recall that OABrowser bookmarks are URL-indexed, globally-readable descriptions of atlas and application state. To store personal bookmarks, an atlas user can log in to the atlas infrastructure using social logins such as Google, Facebook, Twitter, or GitHub. Once logged in, the user can save a bookmark to a Firebase-backed central store. The user can return to the same view of the atlas by accessing a bookmark list in the browser application or by pasting the bookmark’s URL into a web browser (Figure 3). The user who creates a bookmark can also delete it.

Because bookmarks from all atlas users are stored centrally on the network, bookmarks can be exchanged among users (assuming that the atlas data is network-accessible). Any user can load any bookmark (though only the bookmark creator can delete the bookmark) provided they have the bookmark URL. All atlas and bookmark state is immutable, so a bookmark itself cannot be edited (though a bookmark can be used as a basis for a newer bookmark. Bookmark URLs can be exchanged like any other page link (e.g., sent by email or embedded in a web page). Alternatively, bookmarks can be sent to authenticated users inside OABrowser by using a simple message system built into the application.

Bookmarks contain links to the source atlas as well as the viewer’s state. If a bookmark refers to a different atlas (or a different version of the same atlas) than the one that is currently loaded into the viewer, OABrowser will ask the user for permission to load the new atlas.

#### Dynamic Shared Views

**Figure 4:**
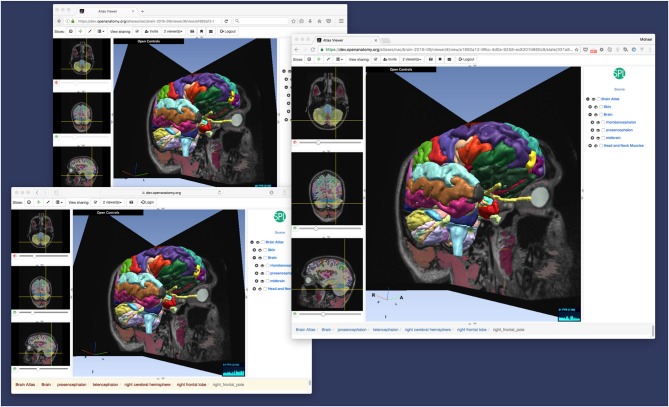
**Dynamic view sharing allows users to view a common, synchronized user interface.** This figure shows OABrowser running in three different web browsers (Safari, Firefox and Chrome) sharing the same atlas view. In this example, the three browsers are running on the same computer, but dynamic views synchronize across any Internet-connected web browsers.

While bookmarks are a useful tool for storing user history and exchanging views of anatomy, OABrowser offers a more interactive mode for interactive collaborative atlas viewing between multiple users: shared dynamic views. A shared dynamic view is like a shared synchronized bookmark whose content can change when users interact with the atlas using the browser. All application state is synchronized between users, and the pointer location of the last user to change the model is also displayed on each remote browser (Figure 4).

This feature can be used to share views between two remote collaborators or a teacher and an entire classroom of students. Dynamic shared views can be exchanged using the application’s messaging system or URLs. In the current implementation, any user sharing a view can manipulate the shared view, which can become confusing with multiple users. In the future, we plan to investigate other collaborative interaction models, such as baton passing or explicit teacher-student mode.

### OABrowser in Use

**Figure 5:**
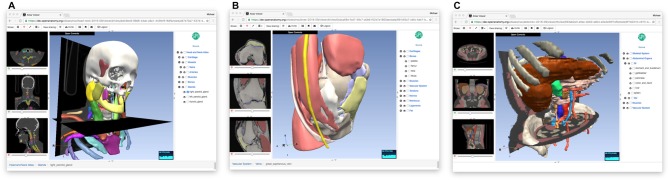
**Other anatomy atlases.** In addition to the SPL/neuroimage analysis center (NAC) Brain Atlas, we have converted three other atlases for use with OABrowser, featuring CT-derived atlases of the head and neck **(A)**, the knee **(B)** and the abdomen **(C)**.

Even as it serves a primary role as a data format test bed, OABrowser has been well-received by our user community. It functions as a lightweight atlas viewer and complements 3D Slicer’s status as a capable but much heavier weight image analysis package in our environment. OABrowser requires no user training for installation and no infrastructure for download, making it suitable for casual use or classroom environments. It provides free access to a handful of locally-developed digital atlases for use in anatomy education and data inspection. Figure 5 shows screenshots of the atlases outside of the brain. Open access is available for both the application source code and the atlas data.

**Figure 6:**
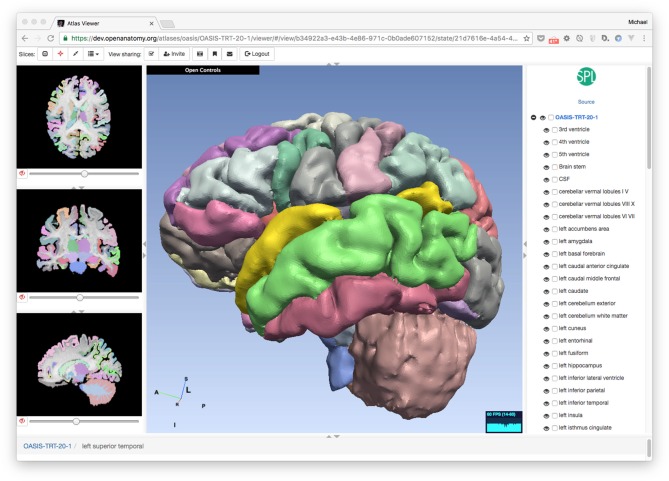
**Converting existing atlases.** OABrowser is designed as a test bed for interoperability between atlases. Here, we show a screenshot of the viewer displaying a version of a segmented brain from the MindBoggle project. We thank Bill Lorensen for his VTK versions of the models. 3D Slicer was used to convert NIFTI files to NRRD.

Though our anatomy atlases are converted from 3D Slicer’s internal data format to HAWG, the new format is simple enough that other source atlases can be converted to it using relatively simple scripting or text editing. In addition to our 3D Slicer converter script, we also provide a python script that can produce an atlas from a text spreadsheet that includes structure names, label values and geometry file names. This approach is perhaps the simplest method of atlas creation. To test interoperability with atlases from other sources, we have converted one of the Mindboggle project’s labeled brain data sets (Klein and Tourville, 2012) to an atlas (Figure 6). Bill Lorensen assisted with the conversion of MindBoggle datasets to VTK as part of his Open Atlas project^18^. The MindBoggle atlas generally displays well in OABrowser, although the initial release of this atlas has misregistration between slices and 3D models. Full compatibility and correct display of community atlases is a high priority for our ongoing work.

### Performance

Interactive performance is an important requirement of any end-user tool. Our current implementation provides adequate performance on a variety of computer platforms. For example, when displaying the SPL/NAC Brain Atlas (which contains 100 of structures and is represented by polygonal models with approximately 500K polygons), the anatomy browser can render image at 60 frames per second on common desktop and laptop computers, and maintain 20 fps on lower-end computers such as Chromebooks and Apple iPad Air 2. As such, we believe that rendering performance of the current application is sufficient for many uses and users.

Loading atlas datasets across the network inevitably produces delays before interaction is possible. OABrowser uses standard web browser caching mechanisms to save local copies of the anatomy atlas data and the viewer application code itself. Reloading atlases thus does not typically require the re-transfer of large data sets. In cases where Internet connectivity may be limited or non-existent, OABrowser’s architecture lends itself to re-packaging as a standalone application that includes multiple anatomy atlas data sets.

## Discussion and Future Work

In this article we have presented OABrower, an experimental anatomy atlas viewer based on developing standards for atlas interoperability. The implementation of OABrowser has allowed us to make changes to early HAWG draft ideas to produce a working, useful atlas browsing tool. Our success in developing OABrowser suggests several different directions for future enhancement of the software and its companion atlases and other tools.

### Implementation Improvements

The current implementation of OABrowser reflects its history as a development test bed and concept demonstration tool. We anticipate several near-term improvements in its implementation to make it both more useful and more maintainable.

At the heart of our future plans for OABrowser is modularization. Several components of OABrowser functionality are independent, capable of being used in other applications and swapped for new implementations. These components include the HAWG parser, the WebGL rendering engine, the image format readers, the bulk data store and cache, the user interface and application core, and the collaborative synchronization mechanism. Modularizing these elements of OABrowser would greatly simplify the community development of the software and encourage new applications with similar features.

In particular, the user interface has not been optimized to work well on mobile platforms: although it runs well enough to demonstrate its promise on phones and tablets, components of the UI are too difficult to access by touch. We intend to both improve the web version of OABrowser using a “mobile first” approach as well as investigate packaging it as a native mobile application using a framework such as Electron^19^.

Modularity will also assist in maintaining compatibility with new versions of the HAWG draft format. Although OABrowser has demonstrated the utility of HAWG’s concepts, we anticipate significant and even incompatible changes before final consensus is reached. A modular HAWG parser shared by several implementations would be extremely useful for encouraging further developments.

Finally, along with changes to the HAWG format comes the need for better and up-to-date conversion tools to help import data into HAWG from other atlas file formats (and when possible export data back). OABrowser’s tools are currently rudimentary and designed for a limited number of data formats. To become a true interoperable data format, the eventual HAWG standard should be consistent, reliable and accessible to the entire anatomy atlas community, including physicians and others who are not computer scientists.

### Editing and Collaboration

OABrowser is primarily a browsing tool, with very limited authoring ability in the form of bookmarks. The current bookmark functionality could be extended with new tools such as region annotation, color selection and text markup. More advanced editors would allow voxel-by-voxel or geometric editing of anatomical segments. Edits to atlases should be transparently committed to version-controlled atlas repositories so that it fits seamlessly into the existing atlas development process.

OABrowser’s web architecture further suggests tighter integration with other browser-based technologies and interaction techniques for new applications. For example, a future version of the viewer could include anatomical tours consisting of lists of bookmarks and accompanying text, audio, or video descriptions by a physician or anatomist. Unlike a traditional slideshow, the view of the anatomy would remain live so that a student could stop at any time and explore a particular anatomic view further. Changes from view to view could animate smoothly to provide visual continuity.

Dynamic shared views represent another opportunity for future enhancement. OABrowser’s current model of view sharing gives all users equal ability to alter the scene. Following a change, the last user to make a change controls the shared pointer. This model of interaction is not suitable for large groups such as classrooms. Better collaborative paradigms such as explicit passing of control could be developed, potentially using lessons learned from teleconferencing applications. Better visual feedback about participants and leaders would also be helpful. If collaborative viewing proved particularly valuable, integrating video technologies such as WebRTC^20^ would allow face-to-face interaction.

### Interoperable Tools

OABrowser and the sample anatomy atlases we have developed are early elements of an interoperable ecosystem of atlas tools and data. By developing an anatomy atlas framework that encourages collaboration and reuse at a variety of levels, we aim to create the largest possible community of developers and users focused on these important repositories of medical knowledge. Interoperability enables some experts to specialize in interactive viewing tools such as AOBrowser. Other teams can focus on atlas editors. Still others can create better annotation tools, or reconcile atlases with biomedical ontologies, or composite multiple atlases into larger virtual ones, or public new data. In an interoperable environment, these research and development efforts can work together with less duplication of effort and more innovation in specific areas. The result is better atlases, with better tools, for new uses and users as well as for existing applications.

The OABrowser viewer is one example of an interactive client for atlas data represented in HAWG format, but other types of clients are similarly enabled by standardization. For example, anatomy atlases can be tested for errors and verified for data consistency by non-interactive testing clients. Other non-interactive clients could, for instance, provide spatial queries against multiple atlases in an atlas library. Once again, such tools could enhance many atlases without requiring direct participation by the atlas creators themselves.

Finally, interoperability suggests a possible alternative, decentralized implementation for a resource such as the Scalable Brain Atlas. Rather than collecting, converting, curating and hosting multiple third-party atlases and building a centralized infrastructure for access and display, a community-driven research group could instead collect references to interoperable atlases and tools (much like NITRC does today). Practical interoperability could be enforced with a test suite of datasets and software tools.

### Atlases as Shared Knowledge

A future environment with high quality interoperable atlas publishing and viewing tools, combined with practical mechanisms for storing, versioning and exchanging atlas data, is one where a rich variety of medical knowledge and experience can be shared. The common current view of anatomy atlases as complete, authoritative and unchanging tomes of information is similar to how encyclopedias were viewed before Wikipedia. Wikipedia has shown that the easier it is to access, publish and share knowledge, the richer and more complete our understanding of the world can be. Anatomical and encyclopedic knowledge are both subject to interpretation, disagreement and revision. Decentralized and streamlined atlas publishing opens new opportunities for the exchange of specialized medical knowledge and the reuse of information for new medical applications.

## Data Sharing and Software Availability

OABrowser-based anatomy atlases are available at https://www.openanatomy.org/. The source code for OABrowser is available on GitHub at https://github.com/mhalle/oabrowser/. The SPL brain atlas is available at https://github.com/mhalle/spl-brain-atlas. OABrowser and the SPL atlases are available under terms of the open-source 3D Slicer license: https://github.com/Slicer/Slicer/blob/master/License.txt

## Author Contributions

MH designed the architecture of OABrowser and currently maintains the code base. VD implemented OABrowser and contributed to its design. RK provided medical and anatomical expertise, conducted testing and used OABrowser for medical education.

## Funding

OABrowser is funded as part of the Atlas Core of the Neuroimage Analysis Center, NIH/National Institute of Biomedical Imaging and Bioengineering (NIBIB) Grant 4P41EB015902; NIH/NIBIB Image Guided Therapy Center (NCIGT) 5P41EB015898; and NIH/National Cancer Institute (NCI) Quantitative Image Informatics for Cancer Research (QIICR) 4U24CA180918.

## Conflict of Interest Statement

The authors declare that the research was conducted in the absence of any commercial or financial relationships that could be construed as a potential conflict of interest. The reviewer CA and handling Editor declared their shared affiliation, and the handling Editor states that the process nevertheless met the standards of a fair and objective review.

## Footnotes

^1^https://www.gnu.org/licenses/licenses.en.html

^2^https://github.com/INCF/HAWG-examples

^3^http://www.spl.harvard.edu/publications/item/view/2037

^4^http://www.spl.harvard.edu/publications/item/view/1953

^5^http://www.spl.harvard.edu/publications/item/view/1918

^6^http://www.spl.harvard.edu/publications/item/view/2271

^7^https://github.com/Slicer/Slicer/blob/master/License.txt

^8^https://angularjs.org/

^9^https://angular-ui.github.io/

^10^https://threejs.org/

^11^http://www.vtk.org/VTK/img/file-formats.pdf

^12^http://teem.sourceforge.net/nrrd/descformat.html

^13^https://www.ietf.org/rfc/rfc4122.txt

^14^https://rethinkdb.com/

^15^https://deepstream.io/

^16^https://git-scm.com/

^17^https://github.com/

^18^https://github.com/lorensen/OpenAtlas

^19^https://electron.atom.io/

^20^https://webrtc.org/
